# The Impact of Economic Crises on Communicable Disease Transmission and Control: A Systematic Review of the Evidence

**DOI:** 10.1371/journal.pone.0020724

**Published:** 2011-06-10

**Authors:** Marc Suhrcke, David Stuckler, Jonathan E. Suk, Monica Desai, Michaela Senek, Martin McKee, Svetla Tsolova, Sanjay Basu, Ibrahim Abubakar, Paul Hunter, Boika Rechel, Jan C. Semenza

**Affiliations:** 1 Norwich School of Medicine, University of East Anglia, Norwich, United Kingdom; 2 Harvard School of Public Health, Boston, Massachusetts, United States of America; 3 Future Threats and Determinants Section, Scientific Advice Unit, European Centre for Disease Prevention and Control (ECDC), Stockholm, Sweden; 4 London School of Hygiene and Tropical Medicine, London, United Kingdom; 5 Department of Medicine, University of California San Francisco, San Francisco, California, United States of America; Finnish Institute of Occupational Health, Finland

## Abstract

There is concern among public health professionals that the current economic downturn, initiated by the financial crisis that started in 2007, could precipitate the transmission of infectious diseases while also limiting capacity for control. Although studies have reviewed the potential effects of economic downturns on overall health, to our knowledge such an analysis has yet to be done focusing on infectious diseases. We performed a systematic literature review of studies examining changes in infectious disease burden subsequent to periods of crisis. The review identified 230 studies of which 37 met our inclusion criteria. Of these, 30 found evidence of worse infectious disease outcomes during recession, often resulting from higher rates of infectious contact under poorer living circumstances, worsened access to therapy, or poorer retention in treatment. The remaining studies found either reductions in infectious disease or no significant effect. Using the paradigm of the “SIR” (susceptible-infected-recovered) model of infectious disease transmission, we examined the implications of these findings for infectious disease transmission and control. Key susceptible groups include infants and the elderly. We identified certain high-risk groups, including migrants, homeless persons, and prison populations, as particularly vulnerable conduits of epidemics during situations of economic duress. We also observed that the long-term impacts of crises on infectious disease are not inevitable: considerable evidence suggests that the magnitude of effect depends critically on budgetary responses by governments. Like other emergencies and natural disasters, preparedness for financial crises should include consideration of consequences for communicable disease control.

## Introduction

Analyzing the complicated and myriad pathways through which economic crises may have impacted infectious disease transmission is fraught with difficulty. The global economic downturn of the past few years is the result of a financial crisis whose scale is unprecedented in the post-war period. With its proximal origins in overly complex credit instruments [1], the crisis initially led to a tightening of private sector credit, and ultimately the collapse of several financial institutions, sharp increases in public sector debt and declines in global trade, markedly lower and in some cases negative GDP growth, and rising unemployment in many industrialised countries [2].

Although the early signs suggest that a fragile recovery is underway [3], it is clear that recent economic damage, principally inflicted during 2008–2009, has led to severe economic hardship for many governments and citizens across the world. The effects of the financial crisis will almost certainly linger beyond any economic recovery. Inevitably, therefore, concerns have been raised that control of infectious diseases could have been and will continue to be adversely affected by budgetary constraints as well as the social effects of recession [4], [5], [6]. For example, some countries have cut budgets for infectious disease control, risking disruption of treatment and/or the exacerbation of drug-resistance [7]. Pharmaceutical companies report declines in sales of prescription drugs, especially in countries with high reliance on out-of-pocket spending [8]. Workers have been reluctant to take sick days, fearing unemployment while increasing the risk of disease transmission at work [9].

Marked rises in infectious disease incidence during previous economic crises and downturns raise concerns about the current situation. During the 1990s, countries of the former Soviet Union (FSU) and Eastern Europe experienced a devastating economic crisis, as GDP fell by one-third on average. Concurrently, the incidence, prevalence and mortality of tuberculosis rose markedly, and worsening treatment led to the emergence of drug-resistant strains [10], [11]. HIV also increased from relatively low pre-crisis levels; outbreaks of diphtheria [12] and tick-borne encephalitis [13], [14] and leptospirosis [15] also occurred. These effects outlasted the immediate crisis period; today, some countries from central and Eastern Europe and former Soviet Union have not been able to achieve Millennium Development Goal (MDG) number 6, ‘to halt or reverse the spread of TB and HIV’ [16].

Even in the absence of economic crisis or downturn, infectious diseases disproportionately affect vulnerable groups. In a review of the European literature, this effect could be found in every single EU Member State [17]. A separate study comparing wealth distribution and TB rates across EU Member States demonstrated a strong correlation between income equality and lower TB rates [18]. Thus, health inequalities, whose importance has been thoroughly documented by the WHO Commission on the Social Determinants of Health [19], may be as relevant for communicable diseases as they are for non-communicable diseases.

In order to gain a better understanding of the interrelationships between economic crises and infectious disease, we conducted a systematic literature review of studies examining the impact of previous economic crises on infectious disease burdens; findings from this analysis can inform communicable disease control research and activities during a time of economic turmoil.

It is first important to define what we mean by “economic crisis”. Commentators often adopt implicit definitions that may include falling GDP, drying up of liquidity and rising or falling prices due to inflation or deflation. Where quantitative measures are used they are applied inconsistently. There is greater clarity in respect of some related terms , which themselves are often treated synonymously with the term “economic crisis”. Thus, a recession is conventionally considered by most politicians and media commentators to exist when there is decline in GDP in two successive quarters but this originated in a simple “rule of thumb” in an article in the New York Times, while others define a recession in terms of increases in unemployment. Academic researchers are more likely to use the National Bureau of Economic Research (NBER) definition of “a significant decline in economic activity spread across the economy, lasting more than a few months, normally visible in real GDP, real income, employment, industrial production, and wholesale-retail sales. A recession begins just after the economy reaches a peak of activity and ends as the economy reaches its trough” [20]. While this definition has considerable utility, these measures can be somewhat remote from ordinary people. Hence, for the purposes of this review we adopt a broader concept of “crisis” to include a number of situations in which individuals and households suffer marked falls in living standards as a consequence of external factors. Specifically, we looked at:

- events that were clearly identifiable from the papers as financial crises, measured in terms of rapid falls in GDP, increases in unemployment, or reductions in government spending that go beyond routine business-cycle fluctuations. This included events explicitly labelled as financial crises, such as that in East Asia in the 1990s, but also situations characterised by the rapid onset of the changes listed here. It also included situations where economic sanctions were imposed by the international community, as in Serbia, or where other events were worsened by sanctions, as in Cuba.
- major political crises or disruption, where this was known from other evidence, although not always referred to explicitly in the papers, to have led to a financial crisis as described above. Many of these studies captured the Transformational Depression in Eastern Europe in the early-1990s which has yielded considerable evidence [21].
- civil and military conflict, which like political crises are often not described in by researchers in terms of their economic consequences but are known to give rise to financial crises.

Our use of this broad approach is justified by the need to capture as much of the relevant literature as possible as we inferred from an initial scoping review of the literature that we would miss important papers if we defined economic crisis in narrow terms. In our tabulated results we include information on the definition of crisis used in each paper. Since the definitions differed significantly across studies, we confined ourselves to a narrative synthesis of the evidence, and we were unable to carry out a quantitative meta-analysis.

We are aware of the extensive literature on the association between economic fluctuations and mortality overall, and to a lesser extent from specific causes. This literature has produced conflicting results, identifying both pro- and counter-cyclic relationships [6], [22], [23], [24], [25], [26], [27], [28], [29], [30], [31]. However, while we have taken account of it in interpreting the findings we report, most of this research does not look specifically at infectious disease. Furthermore, as infectious diseases now comprise a small proportion of overall mortality in industrialised populations, this research, which has mostly examined changing death rates, would not be expected to find significant effects.

## Methods

Although past economic crises, downturns and situations of major political turmoil (such as the collapse of the Soviet Union) are instructive, each event has its own unique characteristics and generalisations need to be made with caution. There is no *a priori* unit for analysis; a broad range of sweeping conjectures and observations can be made about economic circumstances, which might be seen as confusing, potentially contradictory, and without clear impetus for further research or clear intervention.

Moreover, the risks are dynamic and complex, with potential non-linear effects as new infections can spark exponential rises in secondary cases; brief high-risk exposures in a susceptible sub-population could generate a subsequent wave of infections more generally; or singular cases may die out rather than provoking local epidemics, generating public disdain for “false alarms”. These effects are also social in nature, and often deemed too difficult to attribute causality. Additionally, there are feedback loops arising from policies (such as whether to cut public health budgets), which are themselves dependent on institutional and economic circumstances as well as on the depth of the crisis at hand. The effects are likely to be disease-specific, as the agent-host-environment framework differs between, say, tick-borne encephalitis and tuberculosis. Finally, there are likely to be variable lag effects; in some cases the health consequences of a crisis will be apparent almost at once but in others the crisis may lead to changes in behaviour or material conditions that contribute to increased infections some years later. Clearly, the latter are much more difficult to identify and attribute to an economic event.

### i. Analytical Framework

**Figure 1:**
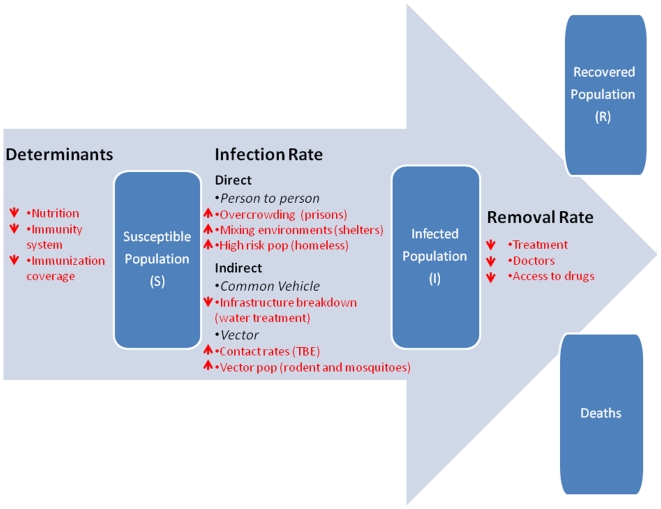
Susceptible, Infectious, Recovered (SIR) Model of Infectious Disease Dynamics. **Notes**: Selected examples of determinants or drivers of ID transmission risk have been added to the SIR Model; however, they do not represent an exhaustive inventory and are intended to visualize potential impacts of the crises. Arrows represent suggested direction of these impacts. Extensions to the basic model for infectious such as TB can account for how factors that will increase progression are different from those that increase infection (as in the SIR model or susceptible, latent, infected TB models); see for example Blower S, McLean, AR, Porco, TC, et al. The intrinsic transmission dynamics of tuberculosis epidemics. Nature Medicine. 1995;1:815–21.

In order to structure the analysis of this study, a version of the classic framework for analyzing infectious disease risks, the susceptible-infected-removed (SIR) model (Figure 1), was employed [32]. This model is a useful intuitive framework for the purpose of organizing our review since it breaks down complex processes into three key analytic parameters. It describes the progress of an infectious disease epidemic by defining those who are susceptible to the disease, those who are infected and those who have recovered and are immune. The transition from ‘S’ (susceptible), to ‘I’ (infected) depends on the transmissibility of the disease and the rate of contact between susceptible and infected persons where ‘I’ is the infectious period, including its determinant, the virulence of the pathogen, which determines the rate of death. Finally, ‘R’ (removed), is the rate of recovery (or death), which is to some degree dependent on the availability of treatments which modify the duration of infectiousness. The rate of transition between these states, *S to I to R*, determines the course of an infectious disease epidemic. This framework can easily be applied to most common childhood diseases but might not be applicable to others (such as gonorrhoea); thus the SIR model is used here solely to frame the discussion rather than to describe the natural history of disease.

Although the SIR model may lack detail for specific diseases (such as durations of latency, or partial immunity, etc.), it does enable an understanding of how an economic crisis could lead to rises (as well as potential reductions) in infectious disease transmission risk. It allows one to break down the complexities of economic crises to pinpoint key components that may be amenable to further study. For example, the SIR model helps to account for some of the dynamics of infectious disease spread in Eastern Europe during the transformational depression of the 1990s:

#### Increasing effective contact rates (S to I)

tick-borne encephalitis rose because it appears that people in the Baltic States spent more time in rural areas, coming into greater contact with ticks [13]; rises in incarceration placed high-risk groups into high-risk transmission conditions for TB, driving an epidemic in Eastern Europe [33], [34].

#### Greater sub-group susceptibility (S to I)

stress related to economic crisis increased high risk behaviour, consumption of alcohol, tobacco, substance abuse, and worse nutrition, all risk factors or the spread of some Ids [35], [36], [37], [38], [39], [40].

#### Reductions in treatment access and quality (I to R)

drops in government spending shift more costs onto patients and lead to disruptions to treatment and reduced quality of care; migration can lead to losses to follow-up [41], [42].

By employing the SIR model in this paper we sought to review studies of infectious disease during periods of economic crisis. Our primary interest and focus was on the experiences in middle and high income countries as those of poor countries raise other issues, some arising from their vulnerability in global markets, others from their very weak health infrastructure, and yet others from the particular burden of disease. Because of marked heterogeneity in the evidence, traditional meta-analytic methods to estimate the hazard of infectious disease spread cannot be applied. Rather, we aimed at identifying key pathways and evidence about the effects of crisis on infectious disease control, including risk of epidemics, emergence and excess deaths. To study the potential for emergence of infectious diseases, we sought to detect changes in the size of high-risk populations and degrees of population exposure to high-risk transmission environments that could be provoked by a crisis. For those additional risks attributable to a crisis, we sought to identify potential public health responses that could mitigate risks of infectious disease epidemics.

### ii. Search Strategy

We first searched the literature for available systematic and narrative reviews that assessed the risks of infectious disease epidemics or emergence due to economic crisis. No systematic reviews were found.

We next searched eight electronic databases for primary studies: Medline, Biosis, Cinahl, ECOnlit, Embase, Global Health, Scopus and Web of Science for - articles registered up to November 2010 (dating back to the initial entries of these enginges, e.g. Medline from 1950, Embase from 1947, etc.; see Table 1). Grey literature databases searched included on-line search engines (Google and Google Scholar) and databases of international organizations (World Health Organization, OECD, European Commission, World Bank, UNAIDS, ECDC and individual country public health institutes websites for European countries) (Table 2). The search terms included “infectious” and “communicable” while, to capture a broad range of effects associated with economic crises and their sequelae, we included the following search terms: economic crisis, fiscal crisis, financial crisis, economic recession, economic depression, economic conditions, economic insecurity, debt, macroeconomic conditions, unemployment, GDP, personnel downsizing, job loss, recession, banking crisis, and business cycle. The complete search strategy is detailed in the Web Annex. Given the complex definitional problems described above, a detailed initial discussion was held among the research team to achieve a shared understanding of the material that would be included, based on the categories of crises identified above.

**Table 1:** Summary of literature on the relationship between economic and political crisis and communicable diseases.

Country	Years examined	Study design	Measure of economic or political crisis	Health Outcome	Key Findings	Source
1. Central and Eastern Europe	1987–1994	Descriptive analysis	Period of economic, political, and social transition	Infectious and parasitic disease mortality	Declining trend in mortality from infectious diseases in Croatia. Stable mortality due to ID in Central and Eastern European countries (50% above EU average)	Hofmarcher, Croatian Medical Journal1998; 39(3): 241–8
2. Central and Eastern Europe	1991–1998	Descriptive analysis	Period of economic, political, and social transition	TB and HIV incidence rate	Increase in incidence of notified TB (34 to 82.1 in Russian Federation, 49.2 to 122.9 in Kyrgyzstan, 55.8 to 114 in Romania, 36.4 to 81.7 in Lithuania)Increase in incidence of HIV infection in IDUs (50–90% increase) in Belarus, Kazakhstan, Moldova, Russian Federation, Ukraine).Increase in syphilis cases from 3000 to 80,000 in UkraineHigh prevalence of MDR-TB (10.7% Estonia, 9% Latvia, 9% Ivanovo, 6.5% Tomsk Oblasts)High incidence of TB in prisons	Migliori, Monaldi Arch Chest Dis 2002; 57(5–6): 285–90
3. Central and Eastern Europe	1989–1991	Descriptive analysis	GNP	Tick-borne encephalitis	Lower GNP associated with higher TBE incidence	Sumilo Reviews in medical virology 2008; 18: 81–95
4. Central and Eastern European countries	1989–1999	Descriptive analysis	Period of economic, political, and social transition	TB	Incidence of TB tripled in Russia in decade after 1991: 34 to 92 per 100,000 persons	Coker et al Lancet 2004; 363(9418): 1389–1392.
5. Central and Eastern Europe	1990s	Descriptive analysis	Period of economic, political, and social transition	Tick-bone encephalitis (TBE)	Increase in incidence of TBE in early 1990s, not seen in neighbouring countries	Randolph 2007; Microbes and Infection, 10: 209–216.
6. Centrally Independent States (former Soviet Union)	1993–4	Descriptive analysis	Period of economic, political, and social transition	Diphtheria	Surge in diphtheria cases to highest incidence of 50,425 in 1995. 1989–90 70% increase in diphtheria cases in Soviet Union1992–93290% increase in Russia alone. Epidemic diphtheria reported from all states except Estonia	Vitek and Wharton; Emerging Infectious Diseases, 1998, (4): 539–550
7. Croatia, East	1991–1995	Descriptive analysis	Period of military conflict	TB	Increase in incidence of childhood TB from rates in 1991 of 4.3 (0–4 years age), 7.6 (5–9 years age), 2.9 (10–14 years age), 23.3 (15–19 years age) to 6.3, 7.6, 12.7, 12.4 respectively in 2003.	Aberle et al. Pediatrics International 2007;49(2):183–9.
8. Czech and Slovak Republic	1993–2002	Descriptive analysis	Period of economic, political, and social transition	Salmonellosis	Increase in salmonellosis cases per 100,000 between 1993 and 2002 from 220.09 to 398.28 in Slovak Republic and 417.65 to 476.4 in Czech Republic	Gulis BMJ 2005; 331: 213–5
9. Eastern Europe and former Soviet Union	1990–1992	Descriptive analysis	Period of economic, political, and social transition	TB	Steady increase in TB mortality in Romania, Armenia, Kyrgystan, Latvia, Lithuania, Moldova, Turmenistan	RaviglioneTubercle and Lung Disease 1994; 75: 400–16
10. Eastern European and former Soviet Union	1992–2002	Multivariate regression	Change in GDP, years of an International Monetary Fund programme, dummy for IMF lending programme, size of IMF loan, urbanization	Tuberculosis	IMF program participation was associated with increases in tuberculosis incidence, prevalence, and mortality by 13.9%, 13.3%, and 16.6%. Decrease in tuberculosis mortality rates of 30.7% associated with exiting the IMF programs.	Stuckler D, King LP, Basu S. PLoS Med 2008;5(7):e143.
11. Estonia	1987–1990 and 1999–2000	Descriptive analysis	Period of economic, political, and social transition	Infectious diseases mortality	Ethnic difference seen between Estonians and Russians- greater increase in deaths due to infectious diseases (and all other causes of death) than ethnic Estonians	Leinsalu J Epi Comm Health 2004; 58:583–89
12. Central and Eastern European countries (n = 15)	1980–2006	Multivariate regression		Excess TB cases and TB mortality	Strong linear association between GDP decline over the period of recession and excess TB cases (r2 = 0.94, p>0.001) and deaths (r2 = 0.94, p<0.001)	Arinaminpathy/Dye Journal of the Royal Society 2010; 7(52): 1559–69
13. European Union countries (26)	1970–2007	Multivariate regression	1% increase in unemploymentMass unemployment (>3% rise)	Age-standardised TB Mortality Rate	No significant effects	Stuckler et al. Lancet 2009; 374 (9686): 315023
14. Germany	1980–2000	Multivariate regression	State unemployment rate	Pneumonia and influenza	1% rise in unemployment rate associated with 3.65% decrease in mortality rate in males, 3.07% decline in both sexes (non-significant for females) in static model. In dynamic model, only significant decline (1.38%) in females. Three times stronger effect in over-65 s.	Neumayer; Social Science & Medicine 2004; 58(6): 1037–47
15. Japan	1950–2002	Descriptive analysis	GDP and national unemployment rate	Pneumonia mortality rate	Negative correlation in both males and females (greater in males than in females) of deaths from pneumonia with unemployment (p<0.001 and p<0.05). No significant association with GDP.	Granados Demography 2008; 45(2): 323–43
16. OECD countries	1960–1997	Multivariate regression	National unemployment rate	Influenza and pneumonia	Deaths from influenza and pneumonia decrease by 1.1% with a 1% increase in national unemployment rate.	Gerdtham Economics and Human Biology 2006; 4(3): 298–316
17. Romania	1990–1998	Descriptive analysis	Period of economic, political, and social transition	Mortality due to AIDS	Increase in mortality in 5–9 years olds due mainly to AIDS	Dolea JECH 2002; 56: 444–9
18. Romania	1980–98 and 1990–2004	Descriptive analysis	War and population migration	Human Trichinellosis	Increase in incidence from 19.6 per 100,000 persons in 1980–89 to 71.8 in 1990–2004	Blaga Am J Trop Med 2007; 76(%):983–86
19. Russia	1988–94, 1999–2000	Descriptive analysis	Post communism economic recession- % decrease in GDP	Infectious and parasitic diseases	Increase in causes of death due to infectious and parasitic diseases in Russia by 9900 cases in 1990–94	Kontorovich 2001; Communist and Post-communist studies
20. Russia	1990–94	Descriptive analysis	Collapse of Soviet Union 1991- decline in average per capita real income by 2/3 between 1990–5	Pneumonia and influenza	Increase in pneumonia and influenza by 135.5%.	Notzon 1998 JAMA
21. Southeast Asia	1990s	Descriptive analysis	Period of East Asian Financial Crisis	HIV/AIDS, STIs	Thailand- 54% reduction in health promotion and 33% reduction in HIV/AIDS budget. Increase in CSW. Increase in AIDS patients reported from 40.89 per 100,000 in 1997 to 43.6 in 1998	Hopkins Health Policy 2006; 75: 347–57
22. Southeastern Europe and Balkans	1963–19681990–95	Descriptive analysis	Transition from communism to capitalism, civil wars	Trichinellosis	Threefold increase in number of cases in Romania 1963–8 v.s. 1969–86	Cuperlovic Veterinary Parasitology 2005; 132: 159–166
23. South Korea	1990–2002	Descriptive analysis	Mortality rates before and after recession (1997) GDP decline from 5–10% to −6.7% in 1998Unemployment rate increase by 5.8% 1997–1999Reduction in household income by 6.7% 1998	Pneumonia and influenza	Age 1–34: Decline in infectious diseases and parasites (1.20 rate ratio 1998/1996 decreased to 0.93 rate ratio 2002/1996 in males and 0.96 decreased to 0.61 in females).Age 35–64: increase in rate ratio between 1998–2000 of approx 40–70%, then decline to ¾ 1996 level by 2002 for pneumonia. Step-wise decrease in TB mortality rates and rate ratio between 1998/1996 and 2002/1996 (0.91 to 0.62 in males and 0.82 to 0.56 in females).Age 65–79: Smaller but still step-wise decrease in TB mortality rates and rate ratio between 1998/1996 and 2002/1996 (0.93 to 0.75 in males and 0.95 to 0.81 in females). Increase in rate of deaths due to pneumonia in males and females between 1998 and 2000 followed by decline.	Khang; Int J Epi 2005; 34: 1291–1301
24. United States	1950–1975	Descriptive analysis	Unemployment variation in United States	Pneumonia and influenza	Little variation in deaths from pneumonia and influenza over this time period. Unemployment only accounts for 1.07% of business cycle variation of death rate.	Eyer Int J Health Services 1977; 7(1): 125–50
25. United States, New York	1974–1995	Cost of illness analysis	Reduction in Department of Health budget by 20%	TB	Closure of 6 of 14 chest clinics52,000 excess cases TBIncrease in drug resistant TB by 13%Increase in homeless population (at increased TB risk) from 7584 to 2349420% excess cases HIVCost of excess TB cases- $500 millionCost of excess HIV cases- $4.7 billion	Freudenberg; Am J Pub Health 2006; 96(30): 424
26. United States, California	2007	Descriptive analysis	Period of housing crisis	West Nile virus	Outbreak of West Nile Virus- 140 cases	Reisen 2008 Emerging Infectious Diseases
27. United States	1980–1982	Econometric study	GNP decline from −0.4 to −1.7, unemployment increase from 7.1% to 9.7%, increase in Consumer Price Index from 88.7 to 288.6	Upper-respiratory problems, genitor-urinary infections, TB, syphilis, Immunization rates	8% suffered from upper-respiratory problems∼7% suffered from genito-urinary infectionsTuberculosis rate increaseCongenital syphilis rose 164%Colorado state reported a 6% drop in the proportion of fully immunized two year olds.The health status and risks for children appeared affected which was attributed to a combination of circumstances that included serious recession, increased poverty rates for households with children and diminished health benefits and social support services.	Miller 1984 World Development Report
28. Uzbekistan	1996	Randomized trial	Period of economic, political, and social transition	Diarrheal diseases	High diarrheal disease rates were caused by drinking water contamination. Deterioration of water treatment and distribution systems resulted in cross-connections between drinking water lines and sewer.	Semenza et al., Am J. Trop. Hyg 1996;59(6)941–946.
29. Cuba	1980–1996	Descriptive analysis	Period of economic decline; U.S. Embargo	Infectious diseases	Decrease in population covered by chlorinated water system from 98% in 1988 to 26% in 1994. Mortality from diarrhoeal diseases rose from 2.7 per 100,000 persons in 1989 to 6.8 in 1993.Outbreak of Guillian Barre secondary to enteric infections in areas that lost chlorination.Rising incidence TB from 5.5 per 100,000 in 1990 to 15.3 in 1994.48% increase in TB deaths between 1992 and 1993.67% increase in infectious and parasitic diseases deaths (8.3 to 13.9 per 100,000)between 1989 and 200377% increase in deaths due to influenza and pneumonia (23.0 to 40.7 percent) between 1989 and 2003	Garfield R, Santana S. American Journal of Public Health 1997;87(1):15–20.
30. Cuba	1989–1995	Descriptive analysis	Period of economic decline	Infectious diseases	Increase in ID proportional mortality in 1989 from 8.3 per 100,000 to 13.4 in 1995.Rates of morbidity due to respiratory diseases, acute diarrhoeal diseases, STIs, infectious neuro, TB (5.1 per 100,000 in 1990 increased to 14.1 in 1995), leptospirosis	Ochoa Int J Health Services 1997; 27(4): 791–807
31. Cuba	1989–1993	Descriptive analysis	Period of economic decline	Diarrhoea, hepatitis A, venereal diseases	Rise in diarrhoea, hepatitis A and venereal diseases	Kuntz 1994 Int J Health Serv.
32. Mexico	1994–1995	Multivariate regression	GDP growth rate	Communicable Diseases	Communicable disease mortality rates increased in the 1994–96 period by 5–6%.	Cutler et al Journal of Public Economics 2002, 84: 279–303
33. Peru	1978–1996	Descriptive analysis	Peru economic crash in late 1980s- GPD fell by 30%, real wages fell by 80%	Malaria, measles, cholera	1.5% of the population infected with cholera in 1991, increases in malaria cases between 1989–1996, measles epidemic 1992 (263 deaths). However unlikely to account for high infant mortality during this period, due to lack of magnitude and timing.	Paxson; World Bank Economic Review 2005; 19(2): 203–223
34. Serbia	1992–1995	Descriptive analysis	UN Economic sanctions, Loss of trade, 31% drop in income	Infectious diseases	Statistically significant effect of war and sanctions (WAS) on mortality due to infectious diseases in men aged 15–24 and 25–34 yrs. Overall male and female mortality from infectious diseases was significantly higher on the basis of the trend for the preceding period.	Vlajinac 1997 J Epidemiol Community Health
35. Serbia	2001–2002	Descriptive analysis	War in former Yugoslavia	Trichinellosis	Rise in trichinellosis incidence	DjordjevicJ Parasitology 2003; 89(2): 226
36. Serbia, Kosovo	1999–2000	Descriptive analysis	Period following military conflict	Tularemia	Outbreak of tularaemia pharyngitis (327 cases)	Reintjes; Emerging Infectious Diseases, 2002, 8: 69–73
37. South Africa	1994	Descriptive analysis	Period of transition to democracy	HIV	Increase in HIV prevalence in South Africa	Parkhurst 2004 Soc Sci Med 59:1913–24

**Table 2:** Grey Literature and commentaries.

Measure of economic or political crisis	Country/setting	Years studied	Health outcome	Study design	Findings	Source
Economic recession in SE Asia	SE Asia	2000	HIV/AIDS	Descriptive trend analysis	Reduction in HIV/AIDS budget by 24% in Thailand in 1998, by 50% in Indonesia in 1999, but no significant changes in morbidity patterns	USAID 2000.
Current economic downturn, 5% decrease in GDP	World	2009	HIV/AIDS	Descriptive trend analysis	Possible interruption of treatment and hence resistance, denial of treatment. Increase in transmission and TB risk as a result.	UNAIDS. The global economic crisis and HIV prevention and treatment programmes: vulnerabilities and impact. Geneva: UNAIDS and World Bank, Global HIV/AIDS program, 2009.
Financial Crisis	Global	2009	Non-specific	Descriptive trend analysis	Recommended action plan for countries in order to mitigate the health impact of the financial crisis.	WHO. Financial Crisis and Global Health: Report of a high-level consultation. Geneva, 2009.
Global Recession	Global	2009	Non-specific	Descriptive trend analysis	Exploratory analysis of the impact of economic crisis on global health and the role that global health actors could play.	The Lancet, 2009, Vol. 373, January 31,The global financial crisis: an acute threat to health. Horton, R.
Financial Crisis	Global	2009	Non-specific	Descriptive trend analysis	The editorial lists the challenges that will be faced by the health sector due to the financial crisis. It covers a wide range of challenges and gives advice to health professionals.	EDITORIAL. Social Medicine, 2009; 83 - Volume 4, Number 2, The Economic Crisis and Public Health, Barry S. Levy, Victor W. Sidel
Financial Crisis	USA	2008	Non-specific	Descriptive trend analysis	Significantly lowered number of dispensed prescription drugs.	Saul S. In sour economy, some scale back on medications. New York Times, October 22, 2008. Available at: http://ww.nytimes.com. Accessed on October 22, 2008.
Financial Crisis	USA	2008	General	Descriptive trend analysis	According to the survey conducted by University of New Hampshire survey centre, 1/3 of Americans had problems paying medical bills and or were unable to afford health care.	2. Lazar K. Medical costs still burden many despite insurance: Mass. survey finds people in debt, skimping on care. Boston Globe, October 23, 2008. Available at: http://www.boston.com. Accessed on October 23, 2008.
Financial crisis	International	2010	Financing for HIV/AIDS policies	Short survey	Concerns regarding the possibility of decreases in HIV/AIDS funding. Examples given are Italy and Ireland who have reduced assistance for global HIV/AIDS programmes	Voelker R. One Casualty of Global Economic Crisis: Uncertain Finances for HIV/AIDS Programs. JAMA 2010; 303(3): 259–61

Inclusion criteria were defined broadly, so as to capture as broad a range of papers as possible initially, but only studies from developed, i.e. high, upper-middle and lower-middle-income nations, as defined by the World Bank [43], were included for the reasons noted above. Qualitative studies, analyses of individual financial markets, and non-English articles were excluded (see Web Annex). Additional exclusion criteria included articles that were qualitative case studies, that did not mention infectious or communicable diseases, and articles not pertaining to economic, financial or political crises (e.g. articles which discussed the impact of poverty on infectious disease transmission, but not in the context of an economic downturn or political crisis).

### iii. Screening of studies and data extraction

The initial database was compiled from the electronic search and duplicates were removed. Two reviewers (MD and MS) screened these citations by title and abstract review. Rare cases of discordance (<0.5%) were resolved by consensus. Relevant citations were organised according to whether they related two key parameters of the SIR model, transmission rate (S to I) or removal rate (I to R). A data entry form was developed and pilot tested in consultation with experts in the area of financial crisis and health. Relevant information from retrieved articles was extracted for a narrative synthesis by two reviewers and summarized in Tables 1 and 2. All selected studies were reviewed by both reviewers and an inter-rater agreement of 100% was obtained for the data.

### iv. Extracting and combining data

We extracted data from each study on the following variables (Web Annex): countries; years examined; study design; measure(s) used to define economic or political crisis; health outcome(s); and key findings. Countries were categorised by level of development, as described above and health effects were categorised into adverse, beneficial, or none. Studies were then mapped onto the SIR model described above.

Substantial differences in methods, data and context of existing studies render a statistical meta-analysis of the findings impossible and limit direct comparison between studies. Ideally, we would have extracted the “effect size” of the impact of – however defined – economic crisis for each study. However, in light of the absence of a common metric for assessing crises, the diversity in methodologies, and in outcome indicators, it was impossible to extract statistically determined effect size estimates that could be combined. For instance, a considerable number of studies merely described in broad terms how infectious disease prevalence or mortality evolved during a period of crisis. Other studies employed a more advanced statistical analysis, even though their “effect size” estimates cannot be considered as estimates of the true causal impact of the crisis. Thus, as we were unable to apply statistical criteria to the studies, our coding of their outcomes was ordinal, assessing whether the authors of a study identified an increase, decrease, or no change in infectious disease incidence, prevalence, and/or mortality rates as reported in the authors' conclusions or the reported models and data.

## Results

We identified 230 unique citations from all literature searches, and of these 37 publications were eligible for inclusion (Table 1). The majority (n = 28; 76%) were descriptive studies, as opposed to analytic studies. The 37 studies were conducted across 27 countries or regions: Out of the 37 studies a total of 30 studies identified adverse infectious disease outcomes, such as an incidence or mortality increases in association with economic crisis; the remaining 7 studies found reductions and/or the absence of a notable, significant effect. In some cases, however, there were also unexpected longer-term benefits of crises, which could not be measured in terms of health outcomes. For example, a rise in infectious disease in Mexico, caused by a deterioration in disease control systems, provided impetus for sweeping healthcare insurance reforms (Seguro Popular) [44].

After observing declining infectious disease mortality in the Great Depression, researchers began to develop ways of conceptualizing and measuring morbidity [45]. There has been speculation that patterns of infectious disease could be influenced by changes in travel and migration, although this is not supported by empirical evidence and the relatively small scale and complexity of any changes, given the now enormous magnitude of global travel, would make research on this issue difficult. Thus, where positive effects were identified, the links were often very tenuous, and the most that can be said is that a crisis can sometimes act as a wake-up call for society to address an issue that would otherwise be neglected.

### i. Transition Rate (Susceptible to Infected)

We found evidence that crises often increased direct and indirect contact rates among human hosts or common vehicles and between human hosts and disease vectors (Figure 1) [13], [34], [46], [47]. We also observed that crises can lead to changes in host behaviour that decrease host immunity [30], [48], [49], [50], [51], [52].

As concerns direct human-to-human transmission, economic downturns may lead to increased crime, especially against property (although this can be mitigated by effective policing [53] and by increased social welfare spending, as occurred in the Great Depression [54]), as well as to increased prison populations. Prisons, in turn, have been shown to act as incubators for tuberculosis, for example, with overcrowding playing a key role, and subsequent spill-overs to the general population [12], [13], [34], [55]. Indirect transmission through a common vehicle was documented in Uzbekistan subsequent to the breakup of the former Soviet Union [56]. High diarrheal disease rates during the summer months were recorded in Nukus, an administrative centre of a region in Uzbekistan. A randomized intervention trial using home chlorination pinpointed the source of these high disease rates: cross-connections between the municipal water distribution system and sewer lines were implicated as the common vehicle in disease transmission. Leaky pipes and lack of water pressure are manifestations of a failing infrastructure, mismanaged during times of economic hardship, which can cross-contaminate the drinking water supply. The political, social and economic upheaval at the time resulted in deterioration of water treatment and distribution systems in Uzbekistan, with serious implications for public health.

Environmental changes in vector habitats may occur due to economic downturns, which could increase contact rates between humans and disease vectors. One study found that mortgage foreclosures in the Californian housing market in California in 2007 caused homes with swimming pools to be abandoned, increasing breeding habitats for mosquitoes. This was linked to an unexpectedly early seasonal increase in West Nile Virus cases [46]. A study of the economic crisis in Kosovo in 1999–2000 found that economic dislocation resulted in the abandonment of food stores. Subsequent rises in rodent populations led to the emergence of tularaemia [47]. An ecologic study suggested that people in Central and Eastern Europe who returned to subsistence agricultural productions, in particular mushroom harvesting, were at greater risk of tick-borne encephalitis [13].

Behavioural changes induced by economic downturns may lead to increased exposure to disease. Loss of income, involuntary unemployment and job insecurity appear to lead to increased tobacco consumption, substance abuse and hazardous drinking, all of which could impair immunity [52]. For example, tobacco use increases the immediate risk of TB mortality and longer-term risk of TB spread and reactivation [35], [51]. Alcohol can increase susceptibility to some infectious diseases, such as pneumonia and tuberculosis [38]. However, some research has suggested that risky behaviours associated with affluent lifestyles can decrease during recessions [27], [57], depending on the price and availability of the substances in question [30], [45]. The implications for infectious disease are not, however, known.

Other factors may also reduce immunity during an economic downturn, but the links are indirect. There is some evidence linking stress to impaired immunological status, by virtue of the cortisol response, increasing susceptibility to certain infectious diseases, [49] [48] although responses to stress vary greatly between individuals [58]. Meanwhile, governments which do not provide food subsidies to indigent populations when faced with rising food prices risk impairing nutrition, a risk factor for several infectious diseases that appears to reflect weakening immunological defences against latent infections, for example, reactivation of tuberculosis [38].

### ii. Recovery Rate (Infected to Recovered)

We found evidence for reduced treatment efficacy and availability; delays prior to accessing therapy arising from reduced detection or longer periods to initiate treatment; development of resistance and reduced treatment adherence; and evidence for higher mortality (albeit reducing the infectious period) (Figure 1).

Much of the evidence relates, on the supply side, to public provision of healthcare services, and on the demand side, to citizen access to the healthcare system. Most of the papers we identified addressed the former. In periods of economic crisis, countries tend to reduce public spending, either to accommodate a tightened fiscal environment, or to adhere to the terms of loans from international donors. Public spending cuts may affect health adversely. One study, for example, suggested that recipients of International Monetary Fund (IMF) loans experienced reductions in public spending (roughly 7.7%), which led to fewer doctors per capita (7.5%) and slower progress in rolling out the WHO-recommended DOTS programme for TB (45%). Overall, loan recipients experienced increases in TB incidence (13.9%), prevalence (13.3%) and mortality (16.6%).

This trend is not only related to nations in receipt of loans. One study reported how closure of health district health centres due to budget cuts during New York City's fiscal crisis in the 1990s led to a failure in follow-up treatment and screening, as well as inadequate availability of front-line drugs. TB incidence rose and the share of reported cases that were multidrug resistant increased from 10% to 25%. Closure of units responsible for HIV control also coincided with significant rises in reported HIV infections, doubling between 1985 and 1990 from 250,000 to 500,000 [52]. One study in Croatia during the 1993 post-war economic recession found that, after the health service closed anti-tuberculosis dispensaries and allocated responsibility for care for tuberculosis to general practitioners, there was an observed increase in diagnostic delays and diminished compliance to TB treatment [59].

Similarly, significant reductions in public sector healthcare provision, especially among low-income groups, during Mexico's financial crisis in 1994–1995 corresponded to a rise in infectious disease mortality rates by 6% [60]. During the South Asian economic crisis of the mid-1990s, there were recessions in both Thailand and Malaysia. However, while the Thai government opted for significant cuts in health expenditure, the Malaysian government increased expenditure on primary care. The Thai government's decision to disinvest in health promotion and HIV/AIDS control was associated with a rise in incidence of AIDS by 2% as well as rises in incidence of STDs and malaria, and a decline in the numbers of immunised children [61]. No such rises were observed in Malaysia at the time, even though numbers of immunized children also decreased.

On the demand side, several factors can lead to reductions in healthcare access by citizenries under financial strain. Public spending on health is vulnerable during an economic downturn; in the USA between 1980 and 1982, reductions in spending were associated with raised incidence of syphilis, tuberculosis, and genitourinary infections as well as diminished immunisation rates among children [62]. Reports further suggest that due to the economic crisis, the poor, especially in countries with high degrees of out-of-pocket spending, are unable to effectively utilize healthcare services and prescribed treatments due to inability to afford co-payments to visit or stay in a health facility or buy prescription drugs [8]. A study of the financial crisis in Peru during the late 1980s reported a 2.5% increase in mortality among children born during this period, attributed to reduced access to health care [63].

Evidence is mixed on the impact of economic downturns on mortality rates. Five studies identified statistically significant associations of rise in unemployment with increases in mortality and morbidity from communicable diseases [27], [57], [63], [64], [65], although the causal mechanisms remain unclear. One study found no association between rises in unemployment rates and infectious disease mortality rates in 26 EU countries over the past three decades [29]. Recent reports from the UK indicate a significant rise in winter deaths in 2008, particularly among the elderly [66], attributed to the financial crisis. In contrast, one study of mortality in Germany demonstrated that a 1% increase in unemployment in Germany had the strongest protective effects on pneumonia and influenza in the population older than 65 years [57]; however, no mechanism was proposed for this counter-cyclical effect.

### iii. Re-introduction rates

We found evidence that economic crisis increased the size of high-risk subpopulations and the risk of “super-spreading environments” among such groups, which may include, among others, homeless populations, ethnic minorities, migrants (especially minors), prison inmates, and the poor. Alongside environmental factors such as crowded living or working conditions, susceptibility among these groups may be heightened by factors such as lower immunity, deteriorated nutrition, or obstructed access to preventative health care.

Several studies found economic hardship increased the population share of high-risk groups. The study of TB incidence during New York City's crisis in 1975 found that the number of homeless people rose by 60%, a group at very high risk of TB [67]. Overall, risks of contracting TB were greatest among those who had experienced reductions in income, involuntary unemployment, and were unable to afford health care [55], [68].

Another high-risk group whose membership tends to increase during economic downturns are migrants, who tend to be at increased risk of infectious diseases and below average access to healthcare services, although there is a great diversity among different migrant groups and their vulnerability is also influenced by their demographics, socio-economic status, social networks, and presence of discrimination as well as disease epidemiology in their country of origin [69]. A different example of large-scale population movements spreading disease occurred in the former Soviet republics, when epidemic spread is thought to have been facilitated by the mass movements of populations [70], especially into prisons [71].

Four studies provided evidence indicating that reduced immunisations among children contributed to infectious disease outbreaks. During the financial crisis in Malaysia in 1996, the number of immunised children decreased substantially as a result of reduced contact with primary care services [61]. Declines in childhood diphtheria immunisations led to the re-emergence of diphtheria in the Former Soviet Union, the first large-scale epidemic in industrialised countries in the past three decades [70]. During an economic recession in the 1980s, the U.S. state of Colorado reported a 6% drop in the proportion of fully immunised two year olds, attributed to increased poverty rates and diminished health benefits and social support services [62].

## Discussion

Assessing the potential impacts of the recent economic turmoil on the spread and control of communicable disease is complicated by the absence of fully analogous historic economic circumstances, not to mention the absence of quantitative studies of disease spread during such downturns. Interpretation of the scientific literature is also complicated by the potential for publication bias. Yet the pathways through which economic havoc could have impacted on communicable diseases in the past, and therefore might do so again in the future, are important and numerous.

Before proceeding to assess the evidence identified in the literature review and narrative synthesis, we must first note several important limitations of the study. First, nearly all studies typically lacked an underlying infectious disease framework (and often the necessary data) for analysing the mechanisms of risk and therefore the potential avenues for intervention. Among those studies reporting reductions in risk, plausible explanations have not been provided, perhaps resulting from this absence of a biological framework [57], [64]. The available research may have missed some longer term consequences. This is inevitable given the myriad of other factors that may intervene over time. Second, there is likely to be some degree of publication bias, although the methodological issues discussed above prevent testing for this, for example by using a funnel plot. Such a bias could skew our findings to identify more negative effects than actually occurred, as null findings or possibly even protective effects may not have been reported to the same extent as negative ones. Third, given the state of existing literature and heterogeneity in outcomes, exposure variables, and methods, we were unable to use quantitative meta-analytic methods to pool data from studies and infer effect sizes. To address these significant limitations of existing studies, we focused on identifying potential effects on infectious disease outcomes of economic downturns that may occur, categories of vulnerable groups, and, where possible assessing the extent to which outcomes may be dependent of identifiable elements of economic and social context.

Acknowledging these important limitations of existing literature, our study provides a first effort to structure existing findings into a theoretical model of infectious disease spread, the SIR model. This framework may serve as a guide for future investigations on economic downturns and infectious disease outcomes. When viewing existing literature through the lens of the SIR model, we found that several studies identified an elevation of risks, both immediately (mainly in terms of increased direct contact rates) and with a delay of several years (in terms of indirect transmission through infrastructure deterioration or reduced health system capacity to provide effective treatment, resulting in longer infectious periods) [4], [72]. These studies provide indications that economic downturns could exacerbate socio-economic inequalities while increasing certain susceptible populations and high-risk spreader groups, such as prisoners, migrants, and the homeless. Lower living standards during times of economic duress may also lead to over-crowding, poorer nutrition and, although the evidence is inconclusive, potentially reduced immunity due to living with higher stress levels. Furthermore, environmental exposures to pathogens may be altered (e.g. subsistence farming or encroachment of wildlife into abandoned areas), creating new opportunities for the spread vector-borne disease. Recovery rates may be similarly affected, with less funding available for healthcare provision, and susceptible groups less able to access healthcare, particularly in health systems that do not offer universal coverage.

These findings provide a cautionary note to policymakers and may facilitate infectious disease control planning in response to current and future economic crises. There is also evidence that the consequences of infectious diseases can also provoke negative economic effects, potentially impeding economic recovery. For example, high HIV burdens have been found to lead to economic instability and poor socio-economic growth prospects in several Sub-Saharan nations, while even relatively limited outbreaks, such as the 2003 SARS outbreak, are estimated to have cost the countries of East and South East Asia some 2% of GDP [73]. Models of the impact of influenza in the UK suggested costs ranging from 0.5% to 1.0% of GDP for low fatality scenarios and 3.3% and 4.3% for high fatality scenarios [74]. This significantly outweighed the economic costs of pre-pandemic vaccines, which were estimated to limit the overall economic impact. Taken together, these studies point to the possibility of a vicious cycle, in which poor health and poor economic growth perpetually undermine one another, either across whole societies or important sub-sets of society.

From a policy perspective, it may be desirable to safeguard and perhaps even bolster infectious disease control budgets during economic downturns. Unfortunately, at a time in which public debt to GDP levels in advanced economies are as high as they have been since the 1950s, and at a time in which population aging is a pressing issue for many countries, the prospects for increasing public health financing are not strong. This may be particularly the case for recipient countries of international aid; Greece, for example, recently accepted European Union and IMF loans after agreeing to undertake rigorous reforms and austerity programmes, including to its health sector [75]. Reforms in and of themselves, of course, can be important opportunities to improve efficiency and service provision within public health systems [5]. However, as we noted earlier, adhering to strict austerity policies may risk a worsening of infectious disease outcomes [76].

Overall, this narrative synthesis highlights the need for better understanding and further quantification of the links between economic downturns and communicable disease spread. This will require research that is highly multi-disciplinary, combining insights from, among others, epidemiology, microbiology, vector biology, ecology, and social geography. Economic downturns may pose increased risks of infectious diseases for some groups in the population. Strategies to identify and engage high-risk groups, while also safeguarding already tight budgets, should be high priorities for the health sector, as communicable diseases, like economic crises, are difficult to control once they begin to spread [77].
